# The Additive Effects of Type-2 Diabetes on Cognitive Function in Older Adults with Heart Failure

**DOI:** 10.1155/2012/348054

**Published:** 2012-06-03

**Authors:** Michael L. Alosco, Mary Beth Spitznagel, Manfred van Dulmen, Naftali Raz, Ronald Cohen, Lawrence H. Sweet, Lisa H. Colbert, Richard Josephson, Joel Hughes, Jim Rosneck, John Gunstad

**Affiliations:** ^1^Department of Psychology, Kent State University, Kent, OH 44242, USA; ^2^Department of Psychiatry, Summa Health System, Akron City Hospital, Akron, OH 44307, USA; ^3^Institute of Gerontology, Wayne State University, Detroit, MI 48202, USA; ^4^Department of Cardiology, Rhode Island Medical Center, Providence, RI 02903, USA; ^5^Department of Psychiatry and Human Behavior, Brown Medical School, Providence, RI 02903, USA; ^6^Department of Kinesiology, University of Wisconsin, Madison, WI 53706, USA; ^7^Department of Medicine, Case Medical Center, University Hospitals, Cleveland, OH 44106, USA; ^8^Harrington Heart & Vascular Institute, Cleveland, OH 44106, USA; ^9^School of Medicine, Case Western Reserve University, Cleveland, OH 44106, USA

## Abstract

*Background*. Medical comorbidity has been theorized to contribute to cognitive impairment in heart failure (HF) patients. Specifically, type-2 diabetes mellitus (T2DM), a common coexisting condition among HF patients, may be an independent predictor of cognitive impairment. Nonetheless, the relationships between T2DM and other risk factors for cognitive impairment among persons with HF are unclear. *Methods*. Persons with HF (*N* = 169, 34.3% women, age 68.57 ± 10.28 years) completed neuropsychological testing within a framework of an ongoing study. History of T2DM, along with other medical characteristics, was ascertained through a review of participants' medical charts and self-report. *Results*. Many participants (34.9%) had a comorbid T2DM diagnosis. After adjustment for demographic and medical characteristics, HF patients with T2DM evidenced significantly greater impairments across multiple cognitive domains than HF patients without T2DM: *λ* = .92, *F*(5, 156) = 2.82, *P* = .018. Post hoc tests revealed significant associations between T2DM and attention (*P* = .003), executive function (*P* = .032), and motor functioning (*P* = .008). *Conclusion*. The findings suggest additive contributions of T2DM and HF to impairments in attention, executive function, and motor function. Future work is needed to elucidate the mechanisms by which T2DM exacerbates cognitive impairment in HF.

## 1. Introduction

Heart failure (HF) affects nearly six million Americans and is associated with elevated rates of mortality [1, 2]. HF is also the most common reason for rehospitalization [3, 4] and is linked to a number of negative outcomes, including poor quality of life [5] and reduced ability to perform instrumental activities of daily living [6, 7].

There is growing evidence that HF is also associated with poor neurocognitive outcomes. Patients with HF are at increased risk for Alzheimer's disease [8, 9], and cognitive impairment is found in as many as 75% patients with HF [10]. Cognitive deficits are associated with functional impairment [11, 12] and increased risk of mortality in this population [13]. Many demographic and medical risk factors for cognitive impairment among older adults with HF have been identified, including older age [14], depression [15], hyperglycemia, history of stroke, elevated systolic blood pressure, metabolic abnormalities (i.e., low serum and anemia) [14], and high plasma brain natriuretic peptide [16], among many others.

Such findings highlight the important role of medical comorbidities in the etiology of cognitive impairment in persons with HF [14]. A condition frequently cooccurring in HF is type-2 diabetes mellitus (T2DM), with as many as 31% of patients with HF also having a comorbid T2DM diagnosis [17]. It is likely that T2DM could be an important contributor to cognitive dysfunction in HF, as patients with T2DM are at increased risk for cognitive impairment independent of HF [18, 19]. One study showed that as many as 18% of community-residing diabetic older adults exhibit cognitive impairment and probable dementia [20]. In addition to specific impairments on neuropsychological tests of attention [21], executive function [22], and processing speed [23], older adults with diabetes also exhibit abnormalities on neuroimaging [23]. Moreover, a recent study demonstrated that when compared to HF patients without T2DM, HF patients with a coexisting T2DM diagnosis have poorer health, reduced quality of life, and shorter long-term survival [24].

Despite these findings, it remains unclear whether T2DM and HF have additive effects on cognitive performance. We examined this possibility in a sample of older adults with HF after adjusting for other important demographic variables and medical comorbidities. Secondary analyses also examined whether T2DM was associated with adverse psychosocial outcomes (i.e., quality of life and activities of daily living) among patients with HF. We hypothesized that HF patients with T2DM would exhibit significantly greater impairments across multiple cognitive domains and have poorer psychosocial outcomes than those without.

## 2. Methods

### 2.1. Participants

A sample of 175 consecutive persons with HF was selected from an ongoing NIH-funded study on cognitive function in HF. After data-screening procedures the sample size was reduced to 169 (see Section 2.4). Participants eliminated were not significantly different from the remaining sample in terms of gender (*χ* ^2^ (1, *N* = 175) = 2.65, *P* = .104), education (*t*(173) = −.75, *P* = .453), depressive symptomatology (as assessed by the BDI-II) (*t*(173) = 1.60, *P* = .170), or history of diabetes (*χ* ^2^ (1, *N* = 175) = .58, *P* = .448). However, those eliminated were significantly younger (*M* = 53.17, SD = 2.32 versus *M* = 68.57, SD = 10.28) than the sample analyzed (*t*(173) = −12.50, *P* = .000).

Participants were recruited from Summa Health System in Akron, OH, USA and reflect the HF population receiving treatment at that facility. The inclusion criteria were age of 50–85 years, English as a primary language, and a diagnosis of New York Heart Association (NYHA) class II or III at the time of enrollment. NYHA history was confirmed by medical record review. NYHA is one of the most commonly used systems to classify symptoms of heart disease and is based on the following scale: (1) mild symptoms with no limitation of physical activity; (2) mild symptoms with slight limitation of physical activity, and ordinary physical activity may result in fatigue, heart palpitations, or shortness of breath; (3) noticeable limitations of physical activity and less than ordinary physical activity results in fatigue, heart palpitations, and shortness of breath; (4) severe limitations of physical activity with symptoms present at rest and increasing discomfort with physical activity [25].

Potential participants were excluded for history of significant neurological disorder (e.g., dementia), head injury with >10 minutes loss of consciousness, severe psychiatric disorder (e.g., schizophrenia and bipolar disorder), substance use, and renal failure. Participants, averaged 68.57 ± 10.28 years of age, were 34.3% female, and 82.8% Caucasian, 11.8% African American, 4.7% Native American, and 0.6% other. See Table 1 for demographic and medical information.

### 2.2. Measures

#### 2.2.1. Neuropsychological Measures

All neuropsychological tests used in the current study have strong psychometric properties, including excellent reliability and validity. The domains and neuropsychological tests administered are as follows: (1) global cognitive function: Modified Mini Mental State Examination (3MS) [26]; (2) attention: Trail Making Test A [27] and Digit Symbol Coding [28]; (3) executive function: Trail Making Test B [29] and Letter Number Sequencing (LNS) [30]; (4) memory: The California Verbal Learning Test-II (CVLT-II) short delay free recall, long delay free recall and total hits [31]; (5) language: Boston Naming Test (BNT) [32] and Animal Fluency [33]; and (6) motor: Grooved Pegboard dominant and nondominant hand [29, 34, 35].

#### 2.2.2. Quality of Life

 The Short Form-12 Quality of Life Measure (SF-12) [36] measures health-related quality of life. The two primary composite scores, Physical Composite Score (PCS; physical functioning, role-physical, bodily pain, and general health) and Mental Composite Scale (MCS; vitality, social functioning, role-emotional, and mental health) were used in the analyses.

#### 2.2.3. Activities of Daily Living

The Lawton Brody Activities of Daily Living Scale were used to assess participants' reported instrumental and basic ADLs. This measure produces a total ADL score with an overall range between 0 and 28, with higher scores indicative of functional independence [37].

#### 2.2.4. Depressive Symptoms

The Beck Depression Inventory-II [38] assessed depressive symptomatology in the current sample. The BDI-II is a commonly used measure with excellent psychometric properties in persons with medical conditions [39]. BDI-II scores range from 0 to 63 with higher scores indicative of increased symptomatology.

#### 2.2.5. Demographic and Medical History

History of T2DM, along with other demographic and medical characteristics, was collected through a review of participants' medical charts and self-report. Specifically, a medical record review was conducted for all participants to corroborate self-report and to ascertain a physician diagnosis of T2DM. Refer to Table 1.

#### 2.2.6. HF Severity

The 2-minute step test (2MST) is an assessment of cardiovascular endurance and was used to serve as an estimate of current heart failure severity [40]. The 2MST requires the patient to march in place for 2 minutes. The patient is asked to bring each knee up to a marked target set on the wall at the individual's own midpoint between the kneecap and crest of the iliac. The number of times that the right knee met the marked target was counted. Higher step count within the 2 minutes was reflective of greater cardiovascular fitness.

The 2MST has been suggested to be an alternative to the 6-minute walk test, which has been linked with functional work capacity, maximal oxygen uptake, and poor prognosis in patients with HF [41, 42]. Recent work has also shown decreased performance on the 2MST to be associated with worse cognitive function in patients with HF [43]. In addition to these findings, the 2MST offers many practical advantages as it is brief, can be conducted within the confines of an examination room, and it is practical for patients with orthopedic devices or poor balance (i.e., patients are permitted to use the wall or a chair for support while performing the 2MST) [40, 44].

### 2.3. Procedures

The local Institutional Review Board (IRB) approved the study procedures, and all participants provided written informed consent prior to study enrollment. Participants completed demographic, medical, and psychosocial self-report measures. A brief neuropsychological test battery was then administered to all heart failure participants to assess attention, executive function, memory, language, and motor functioning. Individuals then completed the 2MST under supervision.

### 2.4. Statistical Analyses

To facilitate clinical interpretation and to avoid undue influence of discrepancy in scales, all raw scores of the neuropsychological measures assessing cognitive function were transformed to t scores (a distribution with a mean of 50, and a standard deviation of 10) using normative data correcting for age. Composite scores for attention, executive, memory, language, and motor functions were means of the t scores within each cognitive domain. Consistent with convention in many clinical settings, impairment in these domains for the current study was defined as a t score of 1.5 standard deviations below the mean (*t* < 35). Screening of the data revealed outliers disrupting univariate normality on executive function, and motor domains, thus these cases were eliminated from analyses (*n* = 6).

To examine the additive effect of T2DM on cognitive impairment, a multivariate analysis of covariance (MANCOVA) was performed using attention, executive, memory, language, and motor functions as the dependent variables. Current medical history of T2DM served as the independent variable for each analysis. Demographic and medical variables including gender, education, depressive symptomatology (as assessed by the BDI-II), heart failure severity (as estimated by the 2MST), and history of stroke, hypertension, myocardial infarction, and elevated cholesterol were all entered as covariates. Age was not included as a covariate because the neuropsychological tests comprising the cognitive domains were corrected for age using normative data. A series of independent samples *t*-tests were then conducted to examine differences between older HF adults with and without T2DM on psychosocial measures assessing quality of life and activities of daily living.

## 3. Results

### 3.1. Demographic and Medical Differences between HF Patients with and without T2DM

 Independent samples *t*-test and *χ* ^2^ statistics were computed to identify differences between HF patients with and without T2DM on important demographic and medical variables. No significant between-group differences were found for age (*t*(167) = −.483, *P* = .629), gender (*χ* ^2^ (1, *N* = 169) = .07, *P* = .798), education (*t*(167) = −1.76, *P* = .080), race (*χ* ^2^ (3, *N* = 169) = 5.967, *P* = .113), or depressive symptomatology (*t*(167) = .99,  *P* = .323). There were also no significant group differences on any of the medical comorbidities, including history of hypertension (*χ* ^2^ (1, *N* = 169) = 3.25, *P* = .072), myocardial infarction (*χ* ^2^ (1, *N* = 169) = .15, *P* = .701), or total elevated cholesterol (*χ* ^2^ (1, *N* = 169) = 1.48, *P* = .224). However, when compared to HF patients without T2DM, HF patients with T2DM performed significantly worse on the 2MST (i.e., evidenced poorer cardiovascular fitness) (*t*(167) = −2.35, *P* = .020). Additionally, although there were no statistically significant between group differences on history of stroke at the *P* < .05 level (*χ* ^2^ (1, *N* = 169) = 1.61, *P* = .204), HF patients without T2DM were nearly twice as likely to have a positive history of stroke than HF patients with T2DM. See Table 1 for details. However, it is important to note that all individuals were carefully screened for the presence of any neurological disorder likely to influence cognitive test performance. As such, patients identified with history of “stroke” were more accurately diagnosed with terms such as “cerebrovascular disease” and “white matter disease” rather than the stereotypic features of a cortical stroke (i.e., loss of language, hemiparesis, etc.).

### 3.2. Cognitive Impairment

T2DM was common in the current sample of HF patients, with 34.9% having a positive medical history of T2DM. Cognitive impairment was also common, as the sample mean 3MS score was 92.79 ± 5.36. Specifically, 24.3% of the participants had a 3MS score below 90, and 39.0% had a 3MS score between 90 and 95 and 36.7% of the sample had a 3MS score between 95 and 100. HF patients with T2DM performed worse on the 3MS than HF patients without T2DM (*t*(167) = −2.02, *P* = .045). HF patients with T2DM had an average 3MS score of 91.66 ± 5.37 (*N* = 59), whereas HF patients without T2DM had an average 3MS of 93.39 ± 5.28 (*N* = 110).

By a T-score cutoff of 35, many HF patients could be classified as impaired on tests of attention, executive function, memory, language, and motor functioning. Impairment in motor functioning (*χ* ^2^ (1, *N* = 169) = 8.85, *P* = .003) was more common in HF patients with T2DM than those without (see Table 2).

### 3.3. T2DM and Cognitive Function in Older Adults with HF

 After adjusting for gender, education, BDI-II, 2MST, and history of stroke, hypertension, myocardial infarction, and elevated cholesterol MANCOVA revealed a significant main effect of T2DM diagnosis across multiple cognitive domains: Λ = .92, *F*(5, 156) = 2.82, *P* = .018. Post hoc tests showed HF patients with T2DM had poorer performance on tests of attention (*F*(8, 160) = 9.29, *P* = .003), executive function (*F*(8, 160) = 4.69, *P* = .032), and motor functioning (*F*(8, 160) = 7.30, *P* = .008). No such pattern emerged for memory (*P* = .635) or language (*P* = .599). See Table 3 for a full summary of group differences in cognitive performance.

### 3.4. T2DM and Performance on Specific Neuropsychological Tests

 Follow-up MANCOVA analyses adjusting for gender, education, BDI-II, 2MST, and history of stroke, hypertension, myocardial infarction, and elevated cholesterol were performed to clarify the relationship between T2DM and performance on neuropsychological tests assessing attention, executive function, and motor functioning. Bonferroni-corrected posttests significance level, set at *P* = .017, revealed that HF patients with T2DM performed significantly worse than those without T2DM on Digit Symbol Coding (*F*(9, 159) = 11.29, *P* = .001), Trail Making Test B (*F*(9, 159) = 6.63, *P* = .011), and Grooved Pegboard with dominant (*F*(9, 159) = 6.34, *P* = .013), and nondominant (*F*(9, 159) = 6.68, *P* = .011) hands.

### 3.5. T2DM and Psychosocial Outcomes in Older Adults with HF

 A series of independent samples *t*-tests revealed group differences in quality of life and ADL function as assessed by the SF-12 PCS (*t*(167) = −2.59, *P* = .011) and total ADL (*t*(99.96) = −2.01, *P* = .048) scores. HF patients with T2DM reported significantly poorer physical quality of life and reduced ability to perform ADLs than HF patients without T2DM. No such pattern emerged on the SF-12 MCS (*t*(167) = −.52, *P* = .605). See Table 4 for details.

## 4. Discussion

 Consistent with past work, cognitive impairment was common in current sample of older adults with HF. Medical comorbidities among patients with HF have been linked with increased risk of cognitive impairment in this population, including hypertension and hyperglycemia [14, 45]. The current study extends these findings by showing that older adults with HF and T2DM have greater impairments in cognitive function than those with HF alone. Such findings suggest that T2DM may be an additional risk factor for cognitive impairment in this population.

 The current study suggests that HF patients with T2DM have additive impairments in cognitive functioning, including attention, executive function and motor functioning. Patients with diagnosis of either HF or T2DM exhibit high rates of cognitive impairment in these domains [10, 17]. Moreover, elevated fasting glucose in clinically normal adults is associated with reduced performance in executive functions [46]. Past work on the etiology of cognitive impairment in persons with HF has identified reduced cerebral perfusion as a result of left ventricular dysfunction [47] and a series of comorbid conditions (e.g., hyperglycemia and stroke history) [14] as important factors. The current findings suggest that T2DM is also an important contributor to cognitive impairment in HF. Such findings are perhaps not surprising given the growing evidence for adverse neurocognitive outcomes in persons with T2DM. For example, poor glycemic control and resulting cerebral microvascular and macrovascular damage (including reduced endothelial functioning) among patients with T2DM have been shown to be associated with cognitive impairment [20, 21, 48–50]. Interestingly, HF is associated with increased risk of Alzheimer's disease (AD) [8, 9], and the high insulin levels often observed in patients with T2DM have also been correlated with greater levels of amyloid-beta protein—an early indicator of AD pathogenesis [51–53]. Future work is needed to clarify the mechanisms by which HF and T2DM may interact.

Follow-up analyses showed that HF patients with T2DM demonstrated greater impairments on a task of psychomotor speed and speeded tasks of attention and executive function. Indeed, past work has shown T2DM patients (independent of HF) to be associated with reduced performance on speeded neuropsychological measures and poorer psychomotor efficiency [54, 55]. Consistent with these findings, patients with T2DM often exhibit a cognitive pattern consisting of decreased mental speed and flexibility [56]. Increased cognitive slowing is an early indicator of cognitive impairment in dementia [57]; thus, future studies should examine whether HF patients with T2DM are at elevated risk for dementia.

The current study found no additive effect of T2DM on memory in older adults with HF. While HF has been linked to impairments in memory, past work suggests that these deficits may be a result of an interaction between older age and low ejection fraction [58]. Similarly, past work has linked T2DM to memory impairment, with poor test performance being closely associated with longer T2DM duration and disease progression [53, 59]. The exact reason for the absence of an additive effect of T2DM on memory in the current sample is unclear. One possible explanation is the absence of direct measures of glycemic control and insulin resistance in the current study. Recent studies suggest that many HF patients exhibit impaired insulin resistance [60] and that this process may occur independent of body composition [61]. As a result, it is possible that participants in the HF-only group of the current study actually exhibit some degree of insulin resistance, and these processes have recently been associated with poorer cognitive function [62]. Future work is needed to clarify this and other possible explanations.

In addition to poorer cognitive function, the current findings also indicate that HF patients with T2DM are at greater risk for reduced functional abilities and poorer quality of life. Such findings are consistent with past work demonstrating HF patients with T2DM to have reduced health-related quality of life [24]. Cognitive impairment may have a profound effect in the treatment management of T2DM in HF patients (i.e., poorer adherence to medication; dietary and physical activity recommendations). For instance, recent work found that cognitive impairment is associated with reduced functional independence in older adults with HF [6], and other work shows greater cognitive impairment among older adults with T2DM is related with poorer disease care management and adherence [63]. Additionally, the current study's findings that HF patients with T2DM reported that a significantly worse physical quality of life, as opposed to mental quality of life, than HF patients without T2DM has significant implications. For example, previous animal studies have shown physical activity to improve insulin resistance and attenuate neuroautonomic dysregulation among rats with cardiovascular disease and HF [64, 65]. Future work should investigate whether physical activity in humans also produces similar metabolic changes and the possibility that such changes would reduce the adverse effects of T2DM on cognitive function in HF patients. Similarly, future studies should also explore the possibility that additive deficits in cognitive function due to multiple comorbidities may be an important cause for adverse psychosocial outcomes in persons with both HF and other common medical conditions in older adults.

Several limitations of the current study merit brief review. First, the present study consisted of cross-sectional data and prospective studies are needed to examine the trajectory of cognitive decline to clarify the relationship of T2DM and cognitive impairment in HF. Additionally, future studies are needed to identify the mechanisms of the adverse effects of T2DM on cognitive function among older adults with HF. Past work suggests cognitive impairment in patients with T2DM to be a result of the negative effects of hyperglycemia and glycation end products on the vascular system, including reduced cerebral blood flow and poorer endothelial functioning [21]. In addition, hyperinsulinemia in T2DM patients has also been linked with cerebral amyloid and tau metabolism [66]. Future studies should examine whether HF patients with T2DM are at greater risk of cognitive impairment through these and other mechanisms.

Finally, the current study examined HF as a broad disease entity, and specific type of HF (i.e., diastolic or systolic) was not examined. While previous research has shown systolic and diastolic HF to both be associated with worse cognitive function [67], the nature by which these HF etiologies interact with T2DM to produce cognitive dysfunction may be distinct. Future work should examine the additive effects of T2DM across HF types. Similarly, the current study examined HF severity using the 2MST, which has been linked with cognitive function in HF persons [43] and also has several practical advantages over commonly used walk tests. However, examining the interaction between T2DM and HF severity using measures such as VO2 max, cardiac output, and/or ejection fraction would provide clearer insight into the effects of T2DM on cardiac functioning. Additionally, future studies should also investigate the contribution of insulin resistance to cognitive function within HF patients using more sophisticated measures (e.g., HbA1C, oral glucose tolerance testing) and to also investigate disease duration. Lastly, the relatively small sample size in the current study did not permit analyses to control for key variables such as medication status and larger samples of HF patients with T2DM that are needed to further elucidate the independent additive effects of T2DM on cognitive function in HF.

In brief summary, the current study demonstrates that HF patients with T2DM have greater cognitive impairments than HF patients without T2DM. Future work is needed to elucidate the mechanisms by which T2DM exacerbates cognitive impairment in older adults with HF.

## Figures and Tables

**Table 1:** Demographic and medical characteristics of older heart failure adults with and without type-2 diabetes mellitus (T2DM).

Demographic characteristics	Heart failure w/o T2DM	Heart failure w/T2DM	Total sample
*N*	110	59	169
Age, mean (SD)	68.85 (11.18)	68.05 (8.42)	68.57 (10.28)
Gender (% women)	33.6	35.6	34.3
Years of education, mean (SD)	13.71 (3.08)	12.87 (2.66)	13.42 (2.96)
Race (% Caucasian)	87.3	74.6	82.8

Medical characteristics			
2MST, mean (SD)	64.14 (11.18)	55.12 (25.72)	60.99 (24.06)
BDI-II, mean (SD)	7.08 (6.83)	8.20 (7.33)	7.47 (7.01)
Stroke (%)	10.9	5.1	8.9
Hypertension (%)	64.5	78.0	69.2
MI (%)	54.5	57.6	55.6
Elevated total cholesterol (%)	63.6	72.9	66.9

Note. 2MST: 2-minute step test; BDI II: beck depression inventory-II; MI: myocardial infarction.

**Table 2:** Cognitive impairment in older heart failure adults with and without type 2 diabetes mellitus.

Cognitive domain	Total sample T score, mean (SD) (*N* = 169)	% <35 T score Total sample (*N* = 169)	% HF w/T2DM T score <35 (*N* = 110)	% HF w/o T2DM T score <35 (*N* = 59)	*χ* ^2^ (*p*)
Attention	48.40 (9.62)	7.7	11.9	5.5	2.22 (.14)
Executive Function	47.36 (11.32)	11.2	15.3	9.1	1.46 (.23)
Memory	45.89 (9.56)	10.7	10.2	10.9	.02 (.88)
Language	51.66 (11.15)	8.3	11.9	6.4	1.53 (.22)
Motor	36.91 (14.18)	34.3	49.2	26.4	8.85 (<.01)

Abbreviations: HF w/T2DM: heart failure with type-2 diabetes mellitus; HF w/o T2DM: heart failure without type-2 diabetes mellitus.

**Table 3:** *T*-score means and standard deviations of cognitive test performance for heart failure patients with and without type-2 diabetes mellitus.

Cognitive domains						
	Attention	Executive function	Memory	Language		Motor
HF w/T2DM (*N* = 110)	44.57 (9.64)	43.81 (13.24)	45.31 (9.15)	50.03 (13.22)		31.87 (14.89)
HF w/o T2DM (*N* = 59)	50.45 (8.99)	49.26 (9.67)	46.20 (9.80)	52.53 (9.82)		39.61 (13.07)
*F*	9.29**	4.69*	.23	.28		7.30**

Performance on neuropsychological tests of attention, executive function, and motor						
	TMTA	Digit	TMTB	LNS	PegsD	PegsND

HF w/T2DM (*N* = 110)	45.60 (13.45)	43.54 (8.23)	38.17 (20.31)	49.45 (8.39)	29.51 (18.54)	34.23 (12.51)
HF w/o T2DM (*N* = 59)	51.17 (10.70)	49.72 (9.19)	47.16 (13.49)	51.38 (9.40)	38.56 (15.76)	40.67 (11.83)
*F*	4.53*	11.29^Ψ^	6.63^Ψ^	.28	6.34^Ψ^	6.68^Ψ^

Note. *= *P* < .05; **= *P* < .01; ^Ψ^= *P* < .017.

Abbreviations: HF w/T2DM: heart failure with type-2 diabetes mellitus; HF w/o T2DM: heat failure without type-2 diabetes mellitus; TMTA: trail making test A; digit: digit symbol coding; TMTBL: trail making test B; LNS: letter number sequencing; PegsD: grooved pegboard dominant hand; PegND: grooved pegboard nondominant hand.

**Table 4:** Means and standard deviations of psychosocial outcomes among older heart failure adults with and without type 2 diabetes mellitus.

	SF-12 PCS	SF-12 MCS	Total ADL
HF w/T2DM	42.10 (8.78)	51.72 (9.64)	24.54 (3.60)
(*N* = 110)			
HF w/o T2DM	45.49 (7.74)	52.54 (9.97)	25.64 (2.93)
(*N* = 59)			
*t* (*df*)	−2.59 (167)*	−.52 (167)	−2.13 (99.96)*

Note. *=*P* < .05.

Abbreviations: HF w/T2DM: heart failure with type-2 diabetes mellitus; HF w/o T2DM: heart failure without type-2 Diabetes mellitus; SF-12 PCS: SF-12 physical composite score; SF-12 MCS: SF-12 mental composite scale; Total ADL: total score on activities of daily living.
